# Autotaxin/lysophosphatidic acid signaling mediates obesity‐related cardiomyopathy in mice and human subjects

**DOI:** 10.1111/jcmm.14005

**Published:** 2018-11-18

**Authors:** Jiakan Weng, Sheng Jiang, Lu Ding, Yi Xu, Xiongfei Zhu, Peifeng Jin

**Affiliations:** ^1^ Department of Cardiac surgery The First Affiliated Hospital of Wenzhou Medical University Wenzhou China; ^2^ Department of Cardiac surgery Sir Run Run Shaw Hospital School of Medicine Zhejiang University Hangzhou China; ^3^ Hubei University of Arts and Science Xiangyang China; ^4^ Department of Pharmacy Wenzhou Medical University Wenzhou China; ^5^ Department of Medicine The Chinese University of Hongkong Hongkong China

**Keywords:** autotaxin, cardiomyopathy, hypertrophy, lysophosphatidic acid, obesity

## Abstract

Obesity is associated with increased cardiovascular morbidity and mortality, but the direct signals to initiate or exaggerate cardiomyopathy remain largely unknown. Present study aims to explore the pathophysiological role of autotaxin/lysophosphatidic acid (LPA) in the process of cardiomyopathy during obesity. Through utilizing mouse model and clinical samples, present study investigates the therapeutic benefits of autotaxin inhibitor and clinical correlation to obesity‐related cardiomyopathy. The elevated circulating levels of autotaxin are closely associated with cardiac parameters in mice. Administration with autotaxin inhibitor, PF‐8380 effectively attenuates high fat diet‐induced cardiac hypertrophy, dysfunction and inflammatory response. Consistently, autotaxin inhibition also decreases circulating LPA levels in obese mice. In in vitro study, LPA directly initiates cell size enlargement and inflammation in neonatal cardiomyocytes. More importantly, circulating levels of autotaxin are positively correlated with cardiac dysfunction and hypertrophy in 55 patients. In conclusion, present study uncovers the correlation between circulating autotaxin and cardiac parameters in mice and human patient, and provided solid evidence of the therapeutic application of autotaxin inhibitor in combating obesity‐related cardiomyopathy.

## 1 INTRODUCTION

The incidence of overweight and obesity in the world has risen dramatically. Obesity is associated with significant metabolic disturbance including hyperlipidemia and may induce cardiovascular diseases.^1^ Among them, cardiomyopathy, exhibiting as cardiac remodelling and dysfunction in terms of hemodynamic load, myocardial inflammation and impaired left ventricular contractility, lead the causes of preventable death in obese patients.^2^ Pathological hypertrophy, usually exhibited as heart failure with reduced ejection fraction is accompanied by adverse cardiovascular events.^3^ Therefore, Better understanding of the underlying mechanisms of pathological hypertrophy might lead to novel therapeutic approaches for alleviating pathological hypertrophy and preventing detrimental outcomes.

Altered cellular metabolism mainly contributes to the development of pathological cardiac hypertrophy. Furthermore, reduced energy production and increased oxidative stress result in cardiomyocyte death and fibrosis.^4^ Abnormal deposit of metabolic intermediates, especially specific lipid intermediates, induces cardiomyopathy.^5^ Obesity‐related cardiomyopathy also considered as lipotoxic cardiomyopathy is frequently observed in obese patients.^6^ Clinical studies show increased fatty acid uptake and oxidation damages the cardiac function in patients.^7^ The accumulation of specific lipid intermediates affects a set of biological process, including mitochondrial function, metabolism and cardiac structure.^8^ Besides other stimuli including natriuretic peptides, non‐coding RNAs and inflammatory cytokines also contributes to the development of cardiac hypertrophy.^9^, ^10^, ^11^ However, how the accumulation of cellular signals to initiate or exaggerate cardiomyocyte hypertrophy remains largely unknown.

Autotaxin is a secreted lysophospholipase D catalyzing the hydrolysis of lysophosphatidylcholine to lysophosphatidic acid (LPA), a pleiotropic growth factor‐like phospholipid.^12^ In humans, increased mRNA expression of autotaxin in fat and elevated circulating autotaxin has also been observed in obese patients with insulin resistance and glucose intolerance compared to those with normal glucose tolerance.^13^ Furthermore, circulating levels of autotaxin correlate positively with insulin resistance in older humans, and are an independent predictor for non‐alcoholic fatty liver disease.^14^ Several insulin resistance‐inducing factors, including interleukin‐6 (IL‐6), glucose and insulin, induce the expression and secretion of autotaxin in inflammatory status.^15^ Consistently, the mRNA expression of fat autotaxin is significantly increased of both dietary and genetic obese mice, which is accompanied by increased circulating level of LPA.^16^ LPA has numerous effects in almost all cell types through its G protein‐coupled receptors, which plays a decisive role in the development of several diseases.^17^ More importantly, a recent study identifies that autotaxin and LPA are involved in the pathobiology of cardiovascular disease.^18^ LPA induces cardiomyocyte hypertrophy via stimulating the activation of Rho‐mediated signals.^19^ However, whether autotaxin/LPA signalling is involved in the process of obesity‐related cardiomyopathy is still unknown.

Present study aims to explore the pathophysiological role of autotaxin/LPA in obesity‐related cardiomyopathy through mouse model and human patients. Utilizing autotaxin inhibitor PF‐8380, we investigate its therapeutic benefits in preventing cardiac hypertrophy and dysfunction in obese mice. Furthermore, the circulating levels of autotaxin are closely correlated with clinical parameters of cardiac function. Overall, our findings suggest autotaxin/LPA signalling mediates obesity‐related cardiac injuries, and administration of specific autotaxin inhibitor is a potential therapeutic approach to improve cardiomyopathy.

## 2 MATERIALS AND METHODS

### 2.1 Reagents

Autotaxin inhibitor PF‐8380 was purchased from Cayman (Cayman Chemical, MI, USA). Mouse autotaxin ELISA kit (#LS‐F16526) was purchased from LSBio (Lifespan Biosciences, WA, USA). Human autotaxin ELISA kit (#DENP20) was purchased from R&D (R&D Systems, MN, USA). The ELISA kits for detecting atrial natriuretic peptide (ANP, #E4349) were purchased from Biovison (Biovison Company, CA, USA). Mouse brain natriuretic peptide (BNP) ELISA kit (#RAB0386‐1KT) was purchased from Sigma chemicals (Sigma, St. Louis, USA). Lysophosphatidic acid ELISA kit (#LS‐F25111) was purchased from LSBio (Lifespan Biosciences). The ELISA kits for detecting NT‐proBNP (#DY3604‐05), TNF‐α (#MTA00B), IL‐6 (#M6000B) and nitric oxide (#KGE001) were purchased from R&D (R&D Systems). Hematoxylin, eosin solution, methylcellulose and 1‐oleoyl (18:1) LPA were purchased from Sigma chemicals (Sigma). Anti‐phosp‐IκB, anti‐IκB and anti‐Tubulin antibodies were from Cell Signaling (Danvers, MA, USA).

### 2.2 Animal experiment

The experimental procedure described here was approved by the Institutional Animal Use and Care Committee at the Wenzhou Medical University (Wenzhou, China). In brief, 6‐week aged male C57BL/6J mice weighing ~20 g were randomly assigned to normal diet (NC) with 12% of calories from fat and high fat diet (HFD, Research diets), providing 60% of calories for 8 weeks. Then the mice were randomly divided into four groups: (a) NC‐fed mice that received 0.5% methylcellulose (NC+Vehicle group); (b) NC‐fed mice that received PF‐8380 (30 mg/kg/day) (NC+PF‐8380 group); (c) HFD‐fed mice that received 0.5% methylcellulose (HFD+Vehicle group); (d) HFD‐fed that received PF‐8380 (30 mg/kg/day) (HFD+PF‐8380 group). These mice were fed STC or HFD for another 8 weeks. The serum and cardiac tissues were collected and stored in −80°C refrigerator before further analysis.

### 2.3 Cardiac histological analysis

Mouse hearts were fixed in 4% paraformaldehyde for 24‐hour and embedded in paraffin. 5 μm paraffin sections were prepared and stained with hematoxylin and eosin solution. To measure the histological changes, the cardiac images were observed under a light microscope (Nikon, Tokyo, Japan).

### 2.4 Echocardiography analysis

Cardiac geometry and functional parameters were recorded in anesthetized mice using echocardiographic examinations which were obtained by using the Vevo 2100 system (Vevo). Anterior and posterior wall thicknesses and diastolic and systolic left ventricular dimensions were recorded from M‐mode images. Echocardiographic parameters included ejection fraction (EF), fraction shorten (FS), left ventricular end‐systolic dimension and left ventricular end‐diastolic dimension.

### 2.5 Cell experiment

Primary mouse cardiomyocytes were isolated from C57BL/6J mice as previously described.^20^ In detail, mouse hearts were removed and perfused with Krebs‐Henseleit bicarbonate buffer containing (in mmol/L) the following: 118 NaCl, 4.7 KCl, 1.2 MgSO_4_, 1.2 KH_2_PO_4_, 25 NaHCO_3_, 10 HEPES and 11.1 glucose. Left ventricles were removed and minced. Hearts were digested with collagenase D for 10 minutes for three times, and the buffer was centrifuged at 500 *g* for 5 minutes. The pallets were suspended and cultured in M199 medium. For LPA treatment, 1 × 10^6^ cells were incubated with different concentration of LPA for 24‐hour.

### 2.6 Total RNA extraction, cDNA synthesis, reverse transcription and real‐time PCR

The total RNA was homogenized in TRIzol and isolated from mouse hearts or primary cardiomyocytes according to the manufacturer's protocol. Reverse transcription was performed with the Superscript III Reverse Transcription System (Invitrogen), and real‐time PCR analysis was performed with SYBR Green (Applied Biosystems, Alameda, CA, USA). The sequence of primers were listed as following: *autotaxin*: F‐GACCCTAAAGCCATTATTGCTAA, R‐GGGAAGGTGCTGTTTCATGT; *ANP:* F‐ACGCCAGCATGGGCTCCTTCTCC, R‐GCTGTTATCTTCGGTACCGGAAG; *BNP:* F‐AAGCTGCTGGAGCTGATAAGA, R‐GTTACAGCCCAAACGACTGAC; β*‐MHC:* F‐AAGTGAAGAGCCTCCAGAGTCTGC, R‐GGGCTTCACGGGCACCCTTAGAGC; *TNF‐*α: F‐GCCACCACGCTCTTCTGTCTA, R‐GATGAGAGGGAGGCCATTTG; *IL‐6*: F‐TCCATCCAGTTGCCTTCTTG, R‐GGTCTGTTGGGAGTGGTATC; *iNOS:* F‐ATGTCCGAAGCAAACATCAC, R‐TAATGTCCAGGAAGTAGGTG; and *GAPDH:* F‐AGGAGCGAGACCCCACTAAC, R‐GATGACCCTTTTGGCTCCAC. Relative gene levels were normalized to *GAPDH* level.

### 2.7 Western blot analysis

Protein extracts (50 μg) from the mouse hearts were boiled for 10 minutes in Laemmli sample buffer and then run on 10% SDS‐PAGE. The protein was then transferred to a polyvinylidene difluoride membrane (Amersham Biosciences). The membrane was blocked for 1‐hour at room temperature with 10% bovine serum albumin in phosphate‐buffered saline/0.05% Tween 20. The blots were incubated overnight at 4°C with anti‐phosp‐IκB, anti‐IκB or anti‐Tubulin antibody and secondary antibody (Cell Signaling). The protein expression was visualized using enhanced chemiluminescence reagents (Bio‐Rad, Hercules, CA, USA). The amounts of the proteins were analyzed using Image J analysis software version 1.38e.

### 2.8 ATX activity assay

Fluorescence FS‐3 was used as substrate to measure ATX activity. Ten microlitre of conditional media was incubated with FS‐3 (5 μmol/L) in reaction buffer (50 mmol/L Tris pH 8.0, 120 mmol/L NaCl, 5 mmol/L KCl, 1 mmol/L CaCl_2_, 5 mmol/L MgCl_2_) in a 96‐well plate for indicated times.^21^ Results were determined by a microplate reader with 488 nm excitation and 520 nm emission.

### 2.9 Human study

We have recruited 55 individuals undergoing cardiac ultrasound measurement at the First Affiliated Hospital of Weznhou Medical University. Exclusion criteria of this study included: patients younger than 18 years or older than 80 years, with known structural heart diseases, congestive heart failure, coronary heart disease, moderate to severe valvular disease, sepsis, electrolyte imbalance, chronic obstructive pulmonary disease, history of liver or renal disease, malignancy, subclinical hyperthyroidism, history of drug abuse or pregnancy. Written informed consent was obtained from all participants and all the procedures were approved by human ethics committee of Wenzhou Medical University.

### 2.10 Statistical analysis

Data were presented as mean ± SD. The Student's *t* test was used for comparing two groups or one‐way ANOVA was used for comparing four groups. GraphPad Prism 5 (GraphPad, San Diego, CA, USA) was used to analyze the statistical significance between sets of data. Differences were considered to be significant at *P* < 0.05.

## 3 RESULTS

### 3.1 Elevated autotaxin level is positively correlated with mouse cardiac hypertrophy

**Figure 1:**
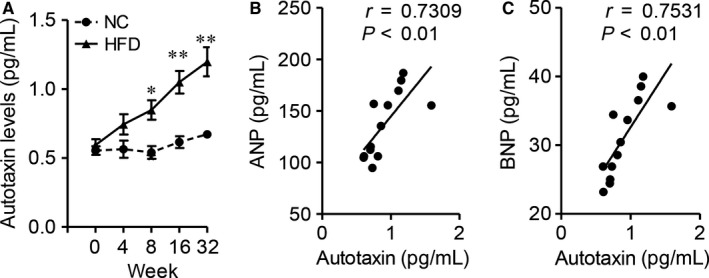
Elevated autotaxin level is positively correlated with mouse cardiac hypertrophy. Male C57BL/6J, aged 6‐week, were fed with normal chow (NC) or high fat diet (HFD) for 0, 4, 8, 16 and 32 weeks. Sacrifice the mice and collect the serum for ELISA analysis. (A) The circulating autotaxin levels. n = 5 mice/group, *P < 0.05, **P < 0.01 as compared with NC‐fed mice. (B and C) Correlation between circulating autotaxin levels and serum ANP (B) or BNP (C) levels. Correlation was assessed by non‐parametric Spearman's test. Results are mean ± SEM

To investigate the pathophysiological role of autotaxin during the obesity, we firstly monitored the circulating autotaxin levels in obese mice. To this end, we fed mice with HFD for 0, 4, 8, 16 and 32‐week, and monitored the changes of serum autotaxin. As Figure 1A showed, HFD time‐dependently increased circulating autotaxin levels, as compared with normal chow (NC)‐fed mice. This result indicated the levels of autotaxin were increased during the obese process. Previous studies have demonstrated that adipose tissues are major places of autotaxin synthesis, and determine the systemic levels of autotaxin.^22^ Here, our real‐time PCR results also found adipose tissue had higher mRNA level of autotaxin in both lean and obese mice (Figure S1).

Atrial natriuretic peptide and BNP, two important secreted factors, are key characteristics of hypertrophic cardiomyopathy.^23^ Therefore, we correlated the levels of autotaxin with these 2 cardiac parameters. As Figure 1B and C showed, circulating autotaxin levels were positively associated with serum ANP (*r* = 0.7309, *P* < 0.01) or BNP (*r* = 0.7531, *P* < 0.01) in mice. These results supported the possible links between autotaxin and obesity‐related cardiomyopathy, especially cardiac hypertrophy.

### 3.2 Autotaxin inhibitor PF‐8380 attenuates HFD‐induced cardiac hypertrophy and function

**Figure 2:**
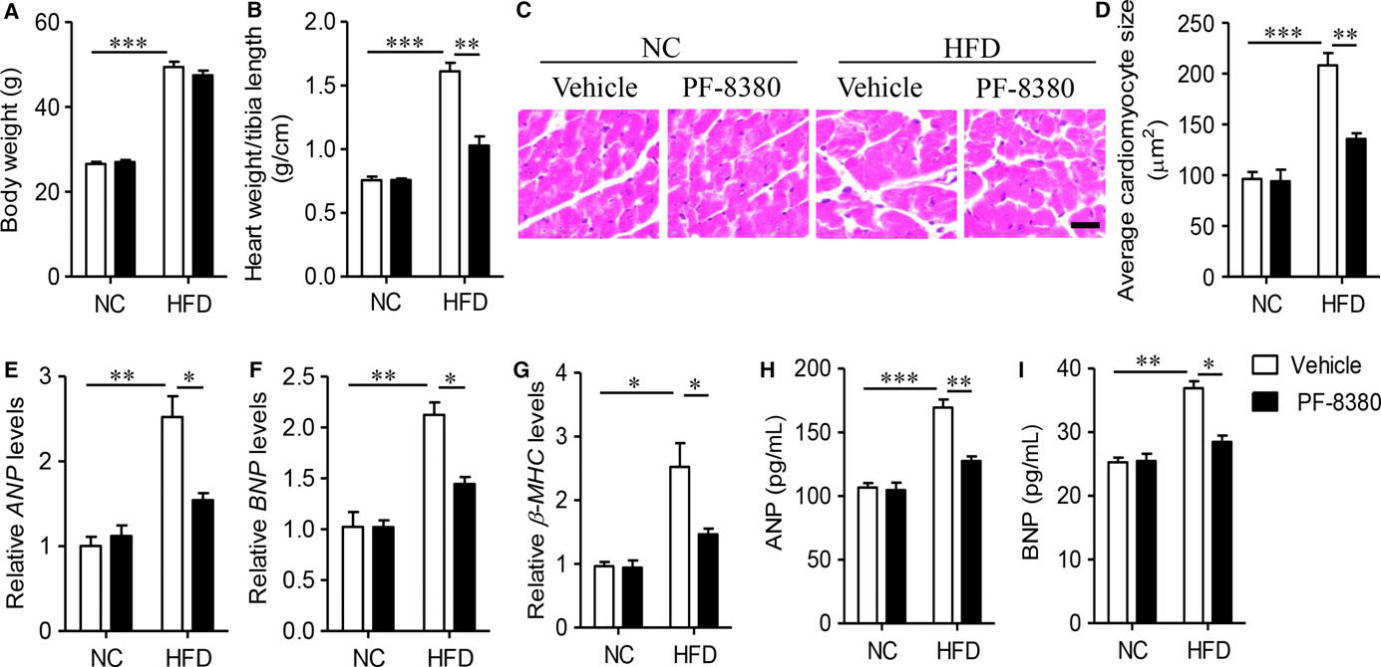
Autotaxin inhibitor PF‐8380 attenuates high fat diet‐induced cardiac hypertrophy. Male C57BL/6J, aged 6‐week, were fed with NC or HFD for 8 weeks, and then treated with vehicle or PF‐8380 (30 mg/kg/day) for 8 weeks. (A) Body weight. (B) The heart weight/tibia length ratio. (C) The histological staining of cardiac tissues by hematoxylin & eosin staining. Scale bar = 40 μm. (D) Quantitative analysis of average cardiomyocyte size. (E‐G) Real‐time PCR measured gene expression of *ANP* (E), *BNP* (F), β*‐MHC* (G) in cardiac tissues. (H and I) ELISA analysis of serum levels of ANP (H) and BNP (I) protein. Results are mean ± SEM, and n = 5 mice/group. *P < 0.05, **P < 0.01, ***P < 0.001

In order to address the role of autotaxin in the obesity‐related cardiomyopathy, we next utilized PF‐8380, a specific inhibitor of autotaxin,^24^ to explore the cardiac changes after suppression of autotaxin activity. Administration of PF‐8380 could not affect HFD‐induced body weight gain (Figure 2A). Then, we measured the ratio of heart weight/tibia length, which is a key marker of cardiac hypertrophy.^25^ As Figure 2B showed, obese mice were significantly increased to 2.1‐fold to NC‐fed mice (*P* < 0.001), but PF‐8380 effectively decreased this increment after 8‐week treatment (*P* < 0.01). The H&E staining showed PF‐8380 significantly attenuated HFD‐induced cardiomyocyte enlargement and structural disorders (Figure 2C and D). Real‐time PCR results also showed PF‐8380 effectively decreased the expression of cardiac hypertrophic genes, including *ANP*,* BNP* and β‐*MHC* (Figure 2E‐G, *P* < 0.05), as compared with vehicle‐treated obese mice. Consistently, autotaxin inhibition also downregulated circulating levels of ANP (Figure 2H, *P* < 0.01) and BNP (Figure 2I, *P* < 0.05).

**Figure 3:**
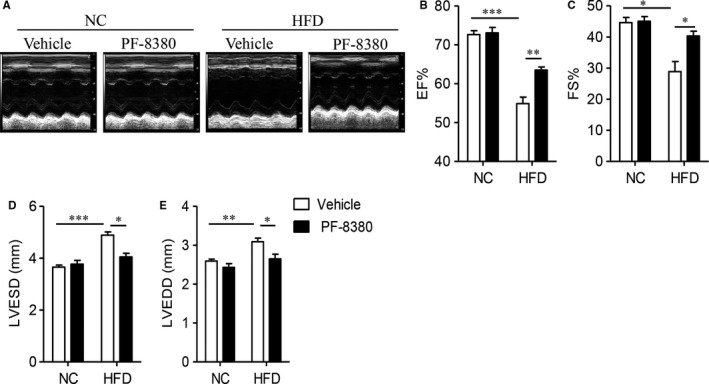
Autotaxin inhibitor PF‐8380 attenuates high fat diet‐induced cardiac dysfunction. Male C57BL/6J, aged 6‐week, were fed with NC or HFD for 8 weeks, and then treated with vehicle or PF‐8380 (30 mg/kg/day) for 8 weeks. (A) Representative images of M‐mode echocardiographic analysis. (B‐E) The measurement of EF% (B), FS% (C), left ventricular end‐systolic dimension (LVESD) (D) and left ventricular end‐diastolic dimension (LVEDD) (E). Results are mean ± SEM, and n = 5 mice/group. *P < 0.05, **P < 0.01, ***P < 0.001

We next evaluated the cardiac performance of these mice using echocardiography. As Figure 3 showed, HFD obviously damaged cardiac function, including reduction of EF (Figure 3B, *P* < 0.001) and FS (Figure 3C, *P* < 0.05). However, administration of PF‐8380 improved cardiac systolic function (Figure 3B and C, *P* < 0.05). Echocardiographic studies also revealed that autotaxin inhibition caused a significant reduction of HFD‐induced left ventricular hypertrophy, including downregulation of systolic and diastolic dimension (Figure 3D and E, *P* < 0.05). All these results supported autotaxin mediated diet‐induced mouse cardiac hypertrophy and dysfunction.

### 3.3 Autotaxin inhibitor PF‐8380 inhibits cardiac inflammatory response in obese mice

**Figure 4:**
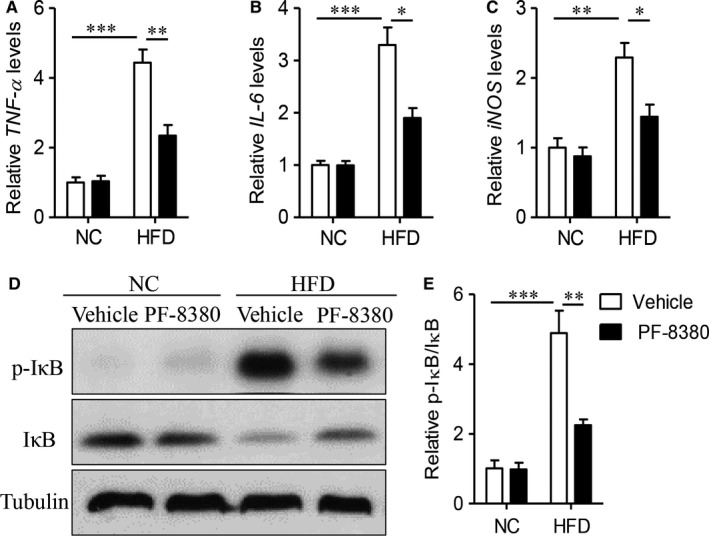
Autotaxin inhibitor PF‐8380 inhibits cardiac inflammatory response in obese mice. Male C57BL/6J, aged 6‐week, were fed with NC or HFD for 8 weeks, and then treated with vehicle or PF‐8380 (30 mg/kg/day) for 8 weeks. (A‐C) Real‐time PCR analysis of inflammatory factors, including *TNF‐*α (A), *IL‐6* (B) and *iNOS* (C) in cardiac tissues. (D‐E) Western blot analysis of protein expression of phosphorylation of IκB and IκB (D), and quantitative analysis of relative density and p‐IκB/IκB ratio (E). Results are mean ± SEM, and n = 5 mice/group. *P < 0.05, **P < 0.01, ***P < 0.001

**Figure 5:**
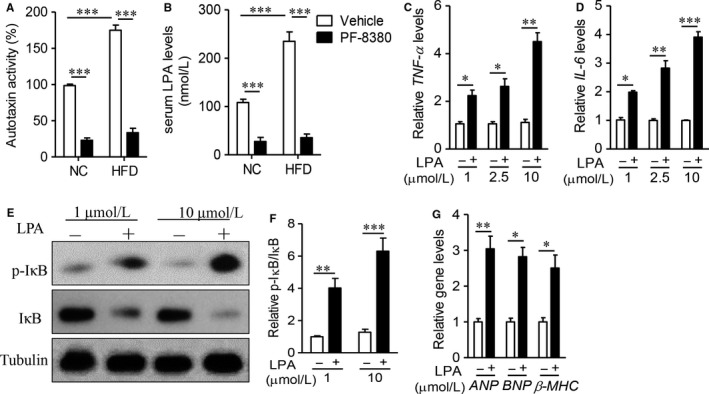
LPA induces cellular damages in primary mouse cardiomyocytes. (A and B) Male C57BL/6J, aged 6‐week, were fed with NC or HFD for 8 weeks, and then treated with vehicle or PF‐8380 (30 mg/kg/day) for 8 weeks. Measurement of autotaxin activity (A) and serum LPA levels (B). (C‐G) The primary mouse cardiomyocytes were isolated from C57BL/6J mice, and cultured in M199 medium. (C and D) Real‐time PCR detected the gene levels of *TNF‐*α (C) and *IL‐6* (D) after autotaxin (1, 2.5 and 10 μmol/L) treatment for 24 h. (E and F) Western blot analysis of protein expression of phosphorylation of IκB and IκB (E), and quantitative analysis of relative density and p‐IκB/IκB ratio (F). (G) Real‐time PCR detected the gene levels of *ANP*,*BNP* and β‐*MHC* after autotaxin (10 μmol/L) treatment for 24 h. Results are mean ± SEM, and n = 5 mice/group. *P < 0.05, **P < 0.01, ***P < 0.001

Cardiac inflammation is a key characteristic of obesity‐related cardiomyopathy, including increasing secretion of inflammatory cytokines and transcriptional activities.^26^ As Figure 4A and C showed, obese mouse had remarkable induction of inflammatory factors, including *TNF‐*α, *IL‐6* and *iNOS* (*P* < 0.01), but administration of PF‐8380 effectively decreased these upregulation in obese mice (*P* < 0.05). On the other hand, PF‐8380 also significantly decreased the levels of systemic inflammatory cytokines, including TNF‐α, IL‐6 and nitric oxide (Figure S2, *P* < 0.05). Nuclear factor (NF)‐κB, as a key transcriptional factor, controls inflammatory response in obesity‐related cardiomyopathy.^27^ As Figure 5D and E showed, PF‐8380 decreased HFD‐induced IκB phosphorylation and degradation (*P* < 0.01), as compared with vehicle‐treated obese mice. Taken together, it demonstrated that autotaxin also controlled diet‐mediated cardiac inflammation in obese mice.

### 3.4 LPA induces cellular damages in primary mouse cardiomyocytes

Previous studies have demonstrated that LPA, produced by autotaxin, regulates monocytosis and promotes inflammation.^28^ To this end, we firstly measured the activity of autotaxin, which controls the production of PLA. As showed in Figure 5A, HFD increased the autotaxin activity to 1.7‐fold, as compared with NC‐fed mice. On the contrast, PF‐8380 significantly decreased the autotaxin activities in both NC‐ and HFD‐fed mice (*P* < 0.001). Consequently, the serum LPA levels were also obviously downregulated in PF‐8380‐treated mice (Figure 5B, *P* < 0.001).

Next, we aimed to investigate the pathophysiological role of LPA in cardiomyocytes. As showed in Figure 5C and D, LPA dose‐dependently increased the gene levels of *TNF‐*α and *IL‐6* in mouse primary cardiomyocytes. In transcriptional level, LPA also activated NF‐κB signalling, including induction of IκB phosphorylation and degradation (Figure 5E and F). Functionally, LPA increased hypertrophic gene levels, including *ANP*,* BNP* and β‐*MHC* in cardiomyocytes (Figure 5G, *P* < 0.05). These results firstly demonstrated the direct injuries of LPA in cardiomyocytes.

### 3.5 Circulating level of autotaxin is closely correlated with human cardiac dysfunction and hypertrophy

**Figure 6:**
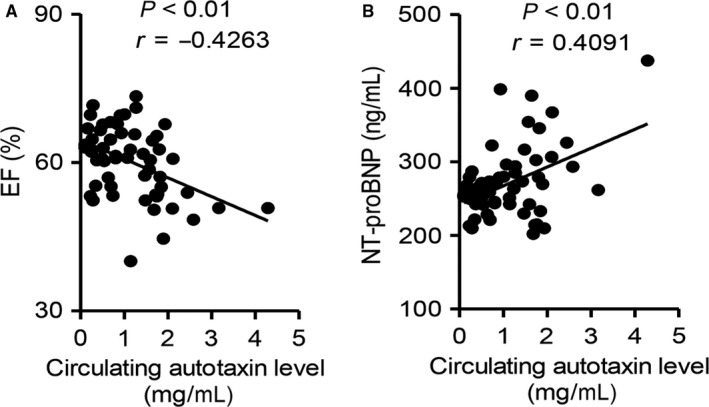
Circulating autotaxin level is closely correlated with cardiac function. Plasma from 55 individuals undergoing cardiac ultrasound measurement were collected and subjected to analysis. Correlation between circulating miR‐29a level and cardiac EF% (A) and plasma NT‐proBNP level (B). Correlation was assessed by non‐parametric Spearman's test

To evaluate the clinical relevance of the circulating autotaxin level and cardiac dysfunction, plasma from 55 patients (age: 45.2 ± 12.2 years; gender: 39 males and 16 females; BMI: 27.5 ± 5.43) were collected. As Figure 6 showed, the serum autotaxin level was negatively correlated with EF value (Figure 6A; *r* = −0.4263, *P* < 0.01), but positively associated with serum level of NT‐proBNP, an important marker of cardiac hypertrophy (Figure 6B; *r* = 0.4091, *P* < 0.01). These clinical results indicated circulating autotaxin was a reliable diagnostic marker for cardiomyopathy.

## 4 DISCUSSION

Present study demonstrates that autotaxin plays a critical role in mediating cardiomyopathy in obese mice. The elevated circulating levels of autotaxin were closely associated with cardiomyopathy, and our results firstly showed autotaxin inhibition obviously improved HFD‐induced cardiac injuries in obese mice. The autotaxin/LPA signalling directly mediated inflammation and hypertrophy in neonatal mouse cardiomyocytes. In human study, the circulating levels of autotaxin were also positively related to cardiac dysfunction. The novel association between autotaxin and diet‐mediated cardiac injuries provides a potential therapeutic target for combating obesity‐related cardiomyopathy.

Previous studies have demonstrated that autotaxin is mainly produced in response to inflammation. For example, the high level of autotaxin persists in association with inflammatory diseases such as arthritis and inflammatory bowel disease.^29^ The production of inflammatory cytokines, such as TNF‐α and IL‐1β, in damaged and inflamed tissue is a signal for increased expression of autotaxin.^30^ In monocytic THP‐1 cells, bacterial lipopolysaccharide, a well‐known initiator of the inflammatory response, induces the expression of autotaxin.^31^ The activity of NF‐κB, a transcriptional regulator of inflammatory cytokines, is closely associated with cellular autotaxin expression.^31^ Consistently, the mRNA expression of fat autotaxin is significantly increased in both dietary and genetic obese mice.^16^ Mechanistically, the upregulation of circulating insulin or glucose levels may induce autotaxin production.^32^ However, whether autotaxin affects inflammatory response or activity is still unknown. Present study found autotaxin inhibitor PF‐8380 suppressed obesity‐induced cardiac inflammation, which supported autotaxin also played feed‐back effects on inflammatory response.

Adipose tissues are major source of circulating autotaxin and other inflammatory cytokines in obese status.^22^ Adipose‐specific deletion of autotaxin causes approximately 40% reduction in circulating LPA, suggests that adipocyte is the major source of circulating autotaxin in mice.^22^ Adipocyte‐specific deletion of autotaxin protects against HFD‐induced metabolic disorders in mouse model.^33^ Similarly, there are already amounts of studies that support the links between heart and peripheral tissues. For example, Adiponectin, an adipose‐secreted adipokine, mediates the modulation of hypertrophic signals in the heart.^34^ Leptin, another adipocyte‐derived 16‐kD peptide, induces hypertrophy in neonatal rat ventricular myocytes.^35^ In our study, specific autotaxin inhibitor effectively improved HFD‐induced cardiac hypertrophy, dysfunction and inflammation in mice. More importantly, circulating levels of autotaxin were significantly correlated with parameters of cardiomyopathy in mice and human patients. All these results support that there is a close crosstalk between adipose tissues and heart, and autotaxin may participate in this tissue interaction.

However, although current study has not addressed the direct or indirect effects of adipose tissue‐derived autotaxin on cardiomyocyte function, our results at least supported circulating autotaxin were closely associated with diet‐induced cardiomyopathy. In future, we will investigate the cardiac changes in specific adipose tissue autotaxin deficient mouse model or mice with fat transplantation, which will solid disclose the tissue interaction.

The pathophysiological roles of autotaxin/LPA signalling in cardiovascular disease have rarely been elucidated now. Recent study shows transported autotaxin and synthesized LPA promotes inflammation and mineralization of the aortic valve.^18^ In in vitro study, LPA stimulates cardiomyocyte hypertrophy, including enlargement of cell size, induction of hypertrophic genes and signalling.^19^ LPA also induces cardiomyocyte hypertrophy through stimulating the expression of microRNA‐23a.^36^ More importantly, LPA significantly stimulates the activity of human BNP promoter,^37^ which determines the cardiac hypertrophy.^23^ These previous findings provide a clue that autotaxin/LPA may be involved in the process of cardiac hypertrophy. Therefore, present study investigated whether autotaxin/LPA signalling is a potential mediator in obesity‐related cardiac hypertrophy. Interestingly, both mouse and human studies found the close correlation between circulating levels of autotaxin and cardiac parameters. Administration with autotaxin inhibitor effectively improved HFD‐induced cardiac dysfunction, hypertrophy and inflammation in obese mice. The upregulated LPA also directly cardiomyocyte inflammation and hypertrophy in vitro. These findings indicate autotaxin/LPA signalling indeed participates in the process of cardiomyopathy in obese condition.

Present study utilized PF‐8380, a specific autotaxin inhibitor, to explore the pathophysiological role of autotaxin/LPA signalling in obesity‐related cardiomyopathy. More importantly, this finding also supported the potential therapeutic benefits of PF‐8380 in protecting against cardiomyopathy. In the past decade, dozens of autotaxin inhibitors have been developed, some of which are now in phase‐II clinical trials for treatment of idiopathic pulmonary fibrosis and breast cancer.^38^ For example, ONO‐8430506, a tetrahydrocarboline derivative with an IC50 of 5 nmol/L for inhibition of plasma autotaxin activity can delay initial tumour growth by 60% in Balb/c mice with orthotopic 4T1 breast tumours and markedly reduced the subsequent lung and liver metastasis.^39^ Therefore, our study suggested autotaxin/LPA signalling was also a potential target for improving cardiomyopathy in obese condition.

In conclusion, our study uncovered the correlation between circulating autotaxin and cardiac parameters in mice and human patients, and provided solid evidence of the therapeutic application of autotaxin inhibitor in combating obesity‐mediated cardiomyopathy.

## CONFLICT OF INTEREST

The authors declare that they have no competing interests.
